# Surgery versus radiotherapy in octogenarians with stage Ia non‑small cell lung cancer: propensity score matching analysis of the SEER database

**DOI:** 10.1186/s12890-022-02177-7

**Published:** 2022-11-10

**Authors:** Lianfang Ni, Gang Lin, Zhigang Zhang, Dan Sun, Zhonghui Liu, Xinmin Liu

**Affiliations:** 1 Department of Geriatrics, Peking University First Hospital, Beijing, 100034 China; 2 Department of Thoracic Surgery, Peking University First Hospital, Beijing, 100034 China

**Keywords:** NSCLC, Octogenarian, Surgery, Radiotherapy, SEER

## Abstract

**Objectives:**

To compare overall survival (OS) and cancer-specific survival (CSS) outcomes of surgery with radiotherapy in octogenarians with stage Ia non-small cell lung cancer (NSCLC).

**Materials and methods:**

Patients aged ≥ 80 years with clinical stage Ia (T1N0M0) NSCLC between 2012 and 2017 were identified from the population-based Surveillance, Epidemiology, and End Results (SEER) database. Patients were assigned into surgery and radiotherapy groups. Multivariate Cox regression analysis was used to identify survival-associated factors. Treatment groups were adjusted by propensity score matching (PSM) analysis while OS and CSS outcomes were compared among groups by Kaplan–Meier analysis.

**Results:**

A total of 1641 patients were identified, with 46.0% in the surgical group and 54.0% in the radiotherapy group. Compared to surgery, radiotherapy-treated patients were older, later diagnosed, had more often unmarried, more squamous cell carcinoma, more unknown grade and increased tumor sizes. Radiotherapy was associated with a significantly worse OS, compared to surgery (hazard ratio 2.426; 95% CI 2.003–2.939; *P* < .001). After PSM, OS (*P* < 0.001) and CSS (*P* < 0.001) were higher in the surgery group. The 1-, 3-, and 5-year OS rates of surgical and radiotherapy group were 90.0%, 76.9%, 59.9%, and 86.0%, 54.3%, 28.0%, respectively. The 1-, 3-, and 5-year CSS rates of surgical and radiotherapy group were 94.5%, 86.1%, 78.0% and 90.7%, 74.5%, 61.0%, respectively. There were no survival differences between the matched surgery without lymph node examination (LNE) and radiotherapy group, as well as between the matched surgery and radiotherapy who were recommended but refused surgery group.

**Conclusions:**

In octogenarians with stage Ia NSCLC, surgery with lymph node dissection offers better OS and CSS outcomes than radiotherapy.

**Supplementary Information:**

The online version contains supplementary material available at 10.1186/s12890-022-02177-7.

## Introduction

Lung cancer is a serious health issue (11.6% of total cancer cases) and the leading cause of cancer-associated death (18.4% of total cancer deaths) [1]. The prevalence of early-stage non-small cell lung cancer (NSCLC) has been projected to increase in the elderly, with the lung cancer screening recommendations and aging of the general population. Elderly individuals are often associated with multiple comorbidities, poor cardiopulmonary functions, frailty, and higher operative risks, which complicates lung cancer treatment [2]. Therefore, it is important to determine the best treatment strategies for octogenarians with early stage NSCLC.

Optimal treatment options for octogenarians and older patients with early-stage NSCLC have not been established. Surgery combined with lymph node dissection is the standard treatment option for early-stage NSCLC [3]. However, due to comorbidities, surgery is often precluded in elderly patients aged ≥ 80 years. Radiotherapy (RT), especially stereotactic ablative radiotherapy (SABR), is the first-line recommendation for inoperable early-stage NSCLC [3]. Over the last decade, SABR has gained an increasing popularity, particularly among older patients. In the United States and European countries, RT has replaced surgery as the most commonly used treatment modality for early-stage NSCLC in patients aged ≥ 80 years [4–6]. However, whether radiotherapy or surgery has a better impact on the treatment for such patients remains unclear. It is difficult to perform randomized clinical trials in octogenarians. In this study, the SEER database was used to compare survival differences between octogenarians and older patients receiving surgery versus those receiving radiotherapy as the sole treatment for stage Ia NSCLC.

## Materials and methods

### Data sources

The SEER database was analyzed via SEER Stat (version 8.3.9; http://www.seer.cancer.gov) in March 2022 with the identifier 15362-Nov2020. SEER 18 Regs Research Plus Data (with additional treatment fields) was chosen to select patients. Its’ follow-up ended by December 31, 2018. This study was conducted in accordance with the Declaration of Helsinki (as revised in Tokyo 2004). Given the retrospective nature of this study anonymization of patient data, patient consent and Institutional Review Board approval were waived.

### Study population

Patients aged ≥ 80 years with histologically diagnosed primary NSCLC in the early stage and treated with surgery or radiation alone from January 2012 to December 2017 were included. Early stage was defined as clinical T1N0M0 (T ≤ 3 cm), classified as stage Ia according to the 8th Edition of the American Joint Committee on Cancer (AJCC) Staging Manual. Patients who had multiple cancers or a second diagnosis of lung cancer, diagnosed by autopsy/death certificate, without sufficient survival data and receiving chemotherapy were excluded.

The following information were obtained for each patient in the SEER database: patient ID, age at diagnosis, sex, race, marital status (unmarried status included widowed, single, divorced and separated), year of diagnosis, anatomical tumor location, size, tumor histology, grade, type of treatment, number of regional lymph node examined (LNE) in patients who underwent surgery, reasons for no cancer-directed surgery in patients who underwent radiotherapy, RX Summ–Surg/Rad Seq and cause of death. Types of treatment categories were: (1) surgery alone, (2) radiotherapy alone. Preoperative or postoperative use of radiotherapy were excluded.

### Statistical analysis

Statistical Analysis were performed using SPSS 26.0 (IBM Corp, Armonk, NY). Patients were assigned into surgery or radiotherapy groups. Survival time was measured from the time of diagnosis to the time of death or last follow-up. The χ^2^ test or Students’t test was used to compare demographic and clinical characteristics while the influence of patient’s characteristics and treatment on OS outcomes were evaluated by Cox regression analysis. Multivariate Cox regression analysis was performed with factors identified as significant in univariate analysis (*p* < 0.05). Propensity score–matched (PSM) analysis was performed based on factors significant in Cox regression analysis for OS. The t-test and χ^2^ test were used to assess the success of propensity score matching. The OS and CSS outcomes before and after PSM were analyzed by Kaplan–Meier (KM) analyses, and evaluated by log-rank test. All tests were 2-sided, with *p* ≤ 0.05 being the threshold for significant differences.

## Results

### Patient characteristics

**Fig. 1:**
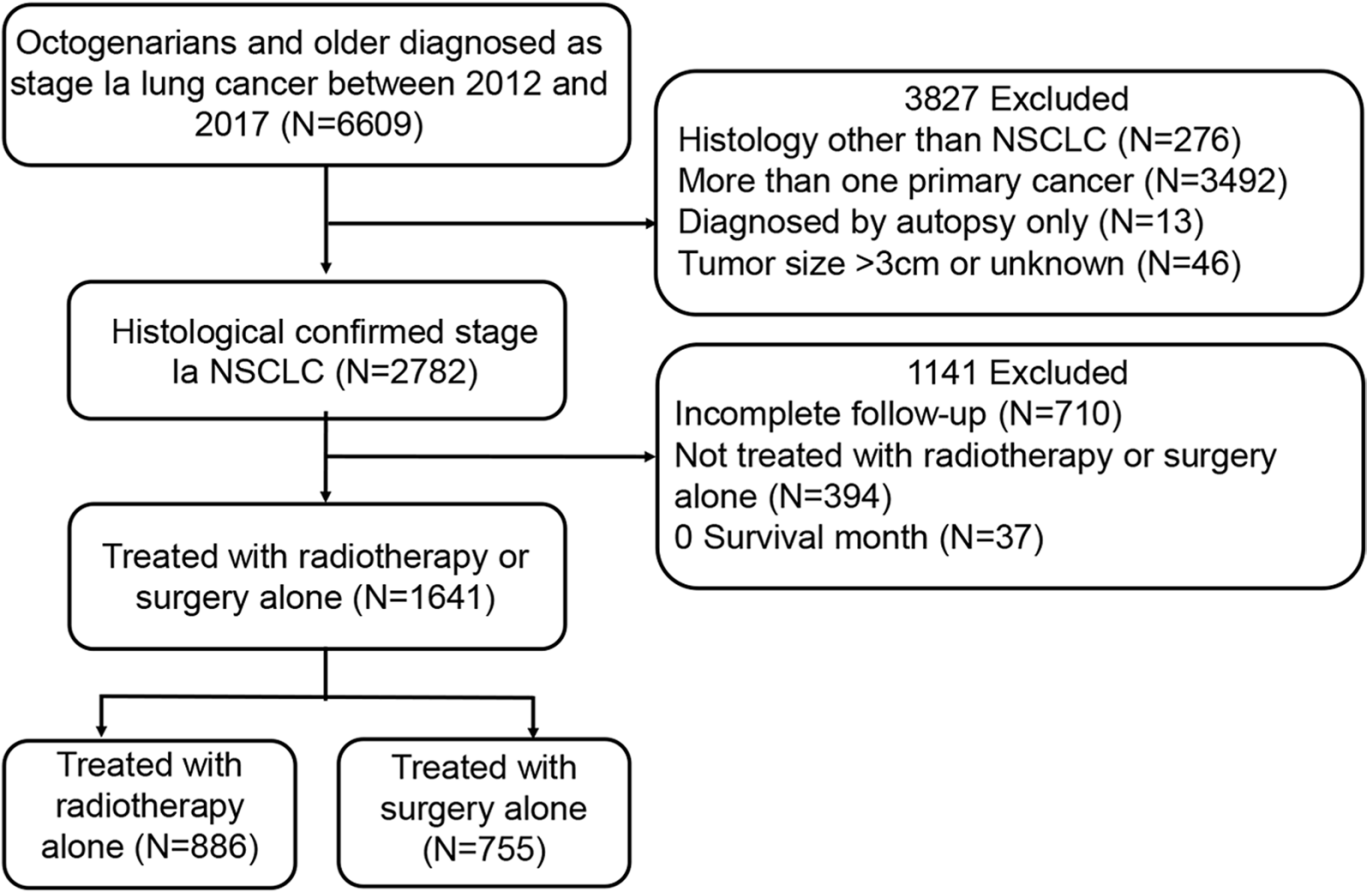
Flow diagram for patient selection

A total of 1641 patients with stage Ia NSCLC were included in this study (755 (46%) subjected to surgery and 886 (54%) subjected to radiotherapy). Figure 1 illustrates the flow diagram for patient selection. The mean age of the entire cohort was 83 ± 2.9 years, while the median follow-up time was 42 months. Baseline demographics and clinical characteristics for surgical and radiotherapy groups are presented in Table 1. Most patients who underwent surgery received either lobectomy (63.4%) or wedge resection (27.5%). Regional LNE was performed in 611 (80.9%) patients in the surgery cohort. Of the 886 patients who received radiotherapy, reasons for no cancer-directed surgery in 808 patients (91.2%) were “not recommended”, while in only 78 (8.8%) patients the reasons were “recommended but not performed, patient refused”. Compared to surgery, patients treated with radiotherapy were older, later diagnosed, and had more often unmarried, more squamous cell carcinoma, more unknown grade and increased tumor sizes.

**Table 1:** Baseline demographics and clinical characteristics of the study participants

Variable	*N* (%)		*p* value
	Surgery (*N* = 755)	Radiotherapy (*N* = 886)	
Age, median ± SD, y	82.6 ± 2.4	84.1 ± 3.1	< 0.001
*Sex*			0.491
Male	298 (39.5%)	335 (37.8%)	
Female	457 (60.5%)	551 (62.2%)	
*Race*			0.149
White	652 (86.4%)	754 (85.1%)	
Black	40 (5.3%)	67 (7.6%)	
Other	63 (8.3%)	65 (7.3%)	
*Marital status*			< 0.001
Married	340 (45.0%)	301 (34.0%)	
Unmarried	379 (50.2%)	549 (62.0%)	
Unknown	36 (4.8%)	36 (4.1%)	
*Year of diagnosis*			< 0.001
2012–2014	393 (52.1%)	364 (41.1%)	
2015–2017	362 (47.9%)	522 (58.9%)	
*Tumor size, cm*			< 0.001
T ≤ 1 cm	69 (9.1%)	35 (3.9%)	
1 < T ≤ 2 cm	373 (49.4%)	379 (42.8%)	
2 < T ≤ 3 cm	313 (41.5%)	472 (52.3%)	
*Histology*			< 0.001
Adenocarcinoma	520 (68.9%)	511 (57.7%)	
Squamous cell carcinoma	159 (21.0%)	304 (34.3%)	
Other	76 (10.1%)	71 (8.0%)	
*Grade*			< 0.001
Well differentiated	201 (26.6%)	98 (11.0%)	
Moderately differentiated	335 (44.4%)	187 (21.1%)	
Poorly or undifferentiated	163 (21.6%)	169 (19.1%)	
Unknown	56 (7.4%)	432 (48.8%)	
*Tumor location*			0.023
Right upper lobe	251 (33.3%)	300 (33.9%)	
Right middle lobe	56 (7.4%)	32 (3.6%)	
Right lower lobe	143 (18.9%)	164 (18.5%)	
Left upper lobe	185 (24.5%)	235 (26.5%)	
Left lower lobe	111 (14.7%)	139 (15.7%)	
Other	9 (1.2%)	16 (1.8%)	

### Survival analysis

**Fig. 2:**
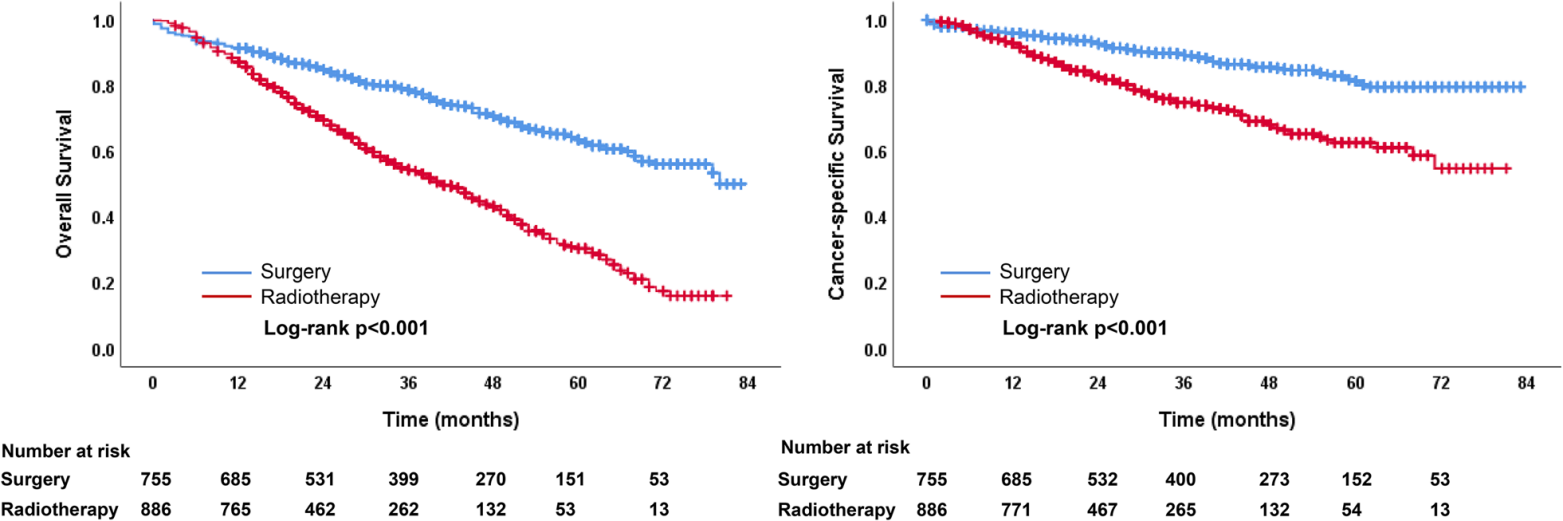
Comparisons of OS and CSS between surgery and radiotherapy groups before PSM

Unadjusted Kaplan–Meier survival curves for unmatched groups are shown in Fig. 2. The median OS time for the entire cohort was 56.0 months (95% CI 51.7–60.3 months). The OS and CSS outcomes of the surgical group were significantly higher than those of the radiotherapy group (both *p* < 0.001). The median OS time was 41.0 months (95% CI, 36.7–45.3 months) in the radiotherapy group while not reached in the surgery group. The 1-, 3-, and 5-year OS rates were 91.4%, 78.6%, and 63.6% in the surgery group, 87.2%, 54.4%, and 30.4% in the radiotherapy group, respectively. The 1-, 3-, and 5-year CSS rates were 95.9%, 89.4%, and 81.4% in the surgery group, and 92.9%, 74.8%, and 62.6% in the radiotherapy group, respectively.

Univariate and multivariate analysis for OS predictors are shown in Table 2. In univariate analysis, age at diagnosis, sex, years of diagnosis, tumor sizes, histology, grades, and treatment patterns were significantly associated with survival outcomes. In multivariate Cox regression analysis, patients who underwent radiotherapy had significantly worse survival outcomes, compared to patients who had surgery (hazard ratio 2.426; 95% CI 2.003–2.939; *p* < 0.001). Younger age, female sex, later year of diagnosis, histology of adenocarcinoma, well or moderate differentiation, and smaller tumor sizes were independent prognostic factors for better OS.

**Table 2:** Cox regression analysis of OS in patients treated with surgery or radiotherapy

Variable	Univariate		Multivariate	
	HR (95% CI)	*p* value	HR (95% CI)	*p* value
Age, y	1.070 (1.042–1.098)	< 0.001	1.029 (1.001–1.058)	0.045
*Sex*				
Male	1.000 (Ref)		1.000 (Ref)	
Female	0.692 (0.592–0.809)	< 0.001	0.712 (0.608–0.835)	< 0.001
*Race*				
White	1.000 (Ref)			
Black	1.184 (0.879–1.595)	0.266		
Others	0.751 (0.543–1.039)	0.084		
*Marital status*				
Unmarried	1.000 (Ref)			
Married	1.094 (0.740–1.617)	0.653		
Unknown	1.009 (0.678–1.501)	0.966		
*Year of diagnosis*				
2012–2014	1.000 (Ref)		1.000 (Ref)	
2015–2017	0.798 (0.669–0.952)	0.012	0.747 (0.625–0.892)	0.001
*Tumor size, cm*				
T ≤ 1 cm	1.000 (Ref)		1.000 (Ref)	
1 < T ≤ 2 cm	1.733 (1.123–2.676)	0.013	1.358 (0.877–2.102)	0.170
2 < T ≤ 3 cm	2.307 (1.499–3.550)	< 0.001	1.615 (1.043–2.500)	0.032
*Histology*				
Adenocarcinoma	1.000 (Ref)		1.000 (Ref)	
Squamous cell carcinoma	1.714 (1.452–2.024)	< 0.001	1.342 (1.121–1.607)	0.001
Others	1.123 (0.854–1.477)	0.405	1.037 (0.782–1.376)	0.799
*Grade*				
Well differentiated	1.000 (Ref)		1.000 (Ref)	
Moderately differentiated	1.496 (1.159–1.931)	0.002	1.268 (0.974–1.652)	0.078
Poorly or undifferentiated	2.136 (1.641–2.779)	< 0.001	1.457 (1.094–1.940)	0.010
Unknown	1.966 (1.525–2.534)	< 0.001	1.055 (0.083–1.387)	0.699
*Tumor location*				
Left upper lobe	1.000 (Ref)			
Left lower lobe	1.075 (0.838–1.378)	0.569		
Right upper lobe	0.988 (0.805–1.212)	0.905		
Right middle lobe	0.777 (0.514–1.177)	0.234		
Right lower lobe	1.087 (0.862–1.372)	0.480		
Other	1.394 (0.775–2.507)	0.267		
*Treatment*				
Operation	1.000 (Ref)		1.000 (Ref)	
Radiotherapy	2.449 (2.073–2.893)	< 0.001	2.426 (2.003–2.939)	< 0.001

**Fig. 3:**
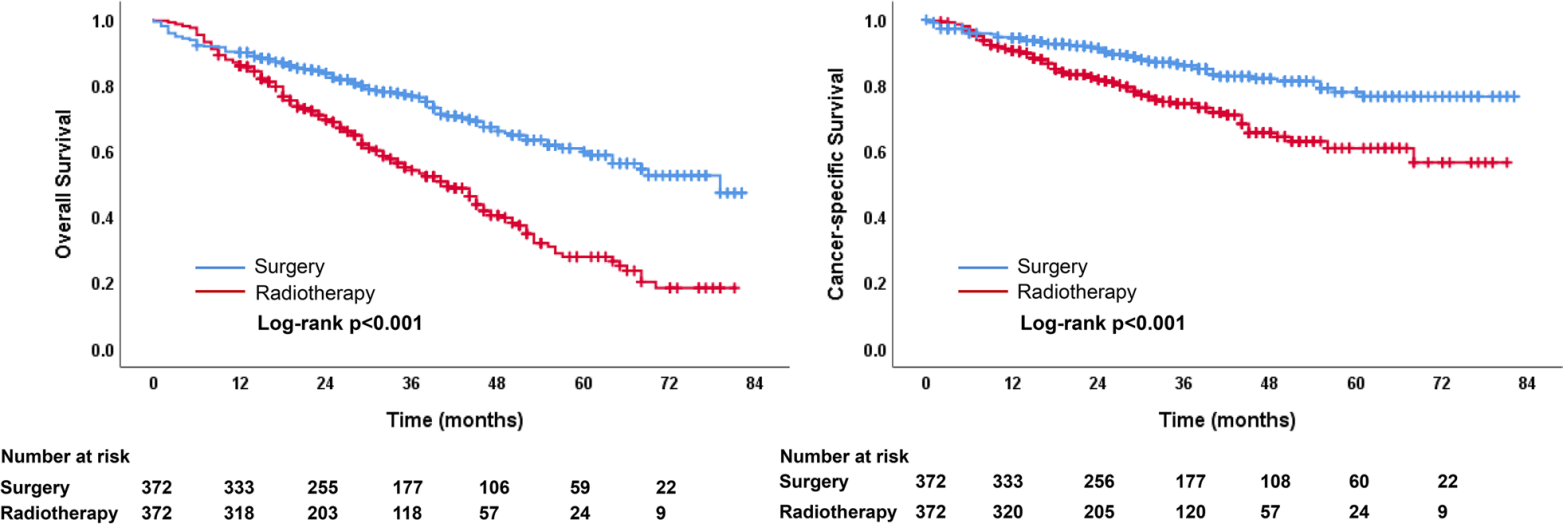
Comparison of OS and CSS between surgery and radiotherapy groups after PSM

Propensity score matching(PSM) based on age, sex, race, year of diagnosis, tumor size, histology, and pathologic grade resulted in 372 patients in both surgery and radiotherapy groups (1:1 ratio). The demographics and clinical variables were well balanced as shown in Tables 3. After PSM, the OS (*P* < 0.001) and CSS (*P* < 0.001) of the surgery group were still higher than that of the radiotherapy group. Median survival time were 79.0 and 41.0 months in the surgery and radiotherapy groups, respectively. The 1-, 3-, and 5-year OS rates were 90.0%, 76.9%, and 59.9% in the surgery group, and 86.0%, 54.3%, and 28.0% in the radiotherapy group. The 1-, 3-, and 5-year CSS rates were 94.5%, 86.1%, and 78.0% in surgery group, and 90.7%, 74.5%, and 61.0% in radiotherapy group, respectively (Fig. 3).

**Table 3:** Characteristics of patients in the surgery and radiotherapy groups after PSM

Variable	*N* (%)		*p* value
	Surgery (*N* = 372)	Radiotherapy (*N* = 372)	
Age, median ± SD, y	83.2 ± 2.5	83.0 ± 2.6	0.208
*Sex*			0.881
Male	150 (40.3%)	148 (39.8%)	
Female	222 (59.7%)	224 (60.2%)	
*Race*			0.514
White	327 (87.9%)	321 (86.3%)	
Black	16 (4.3%)	23 (6.2%)	
Other	29 (7.8%)	28 (7.5%)	
*Marital status*			0.193
Unmarried	193 (51.9%)	214 (57.5%)	
Married	168 (45.2%)	144 (38.7%)	
Unknown	11 (3.0%	14 (3.8%)	
*Year of diagnosis*			0.825
2012–2014	174 (46.8%)	171 (46.0%)	
2015–2017	198 (53.2%)	201 (54.0%)	
*Tumor size, cm*			0.404
T ≤ 1 cm	25 (6.7%)	17 (4.6%)	
1 < T ≤ 2 cm	168 (45.2%)	178 (47.8%)	
2 < T ≤ 3 cm	179 (48.1%)	177 (47.6%)	
*Histology*			0.205
Adenocarcinoma	245 (65.9%)	244 (65.6%)	
Squamous cell carcinoma	97 (26.1%)	109 (29.3%)	
Other	30 (8.1%)	19 (5.1%)	
*Grade*			0.297
Well differentiated	66 (17.7%)	73 (19.6%)	
Moderately differentiated	158 (42.5%)	146 (39.2%)	
Poorly or undifferentiated	98 (26.3%)	87 (23.4%)	
Unknown	50 (13.4%)	66 (17.7%)

**Fig. 4:**
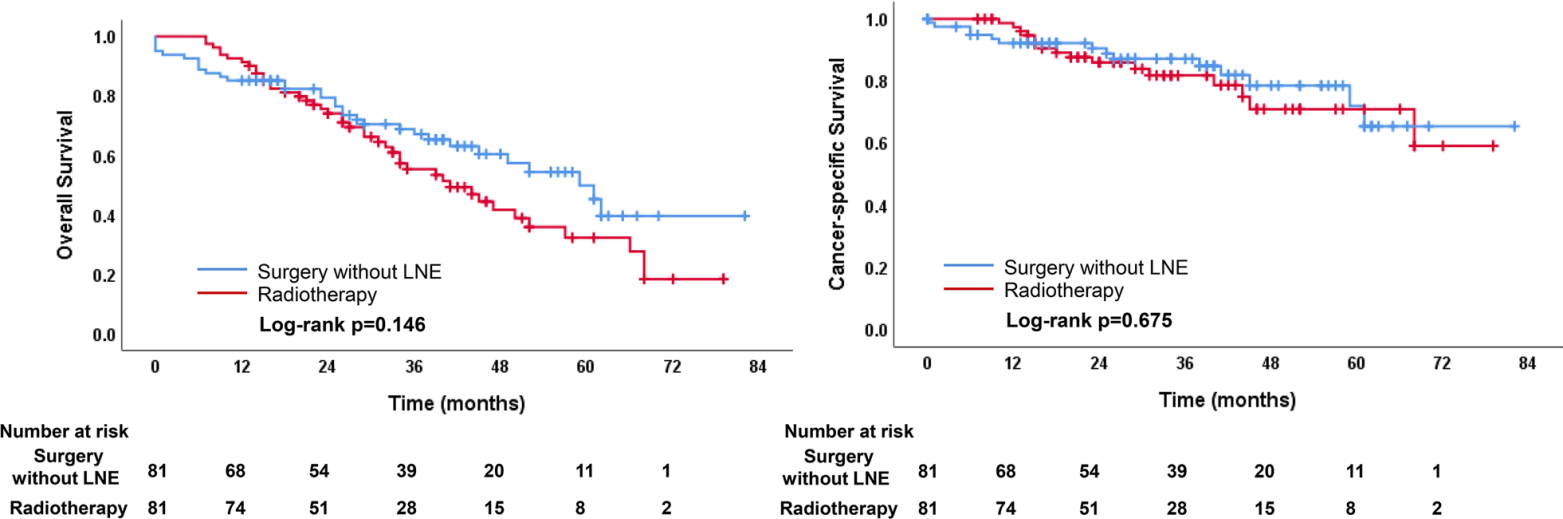
Comparison of OS and CSS between surgery without LNE and radiotherapy groups after PSM

Survival outcomes were compared between matched subgroups of surgery without LNE versus radiotherapy. The demographics and clinical variables were well matched after propensity score matching (Additional file 1: eTable 1). Kaplan–Meier survival curves for the matched groups are displayed in Fig. 4. No differences in the OS (*P* = 0.146) and CSS (*P* = 0.675) were found between the surgery without LNE and radiotherapy group.

**Fig. 5:**
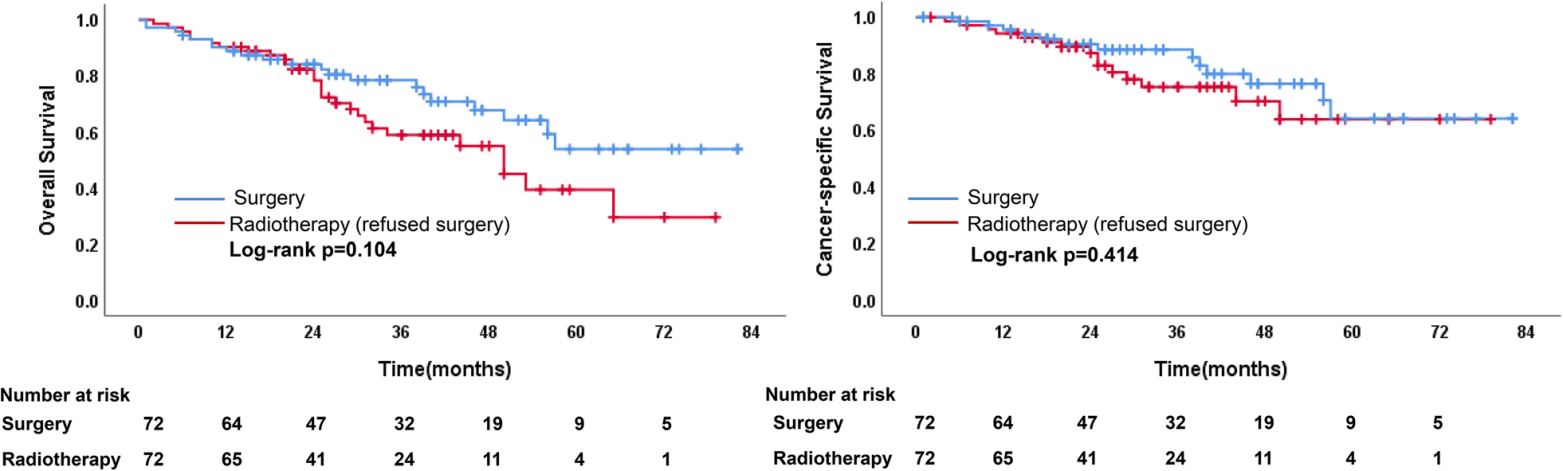
Comparison of OS and CSS between surgery and radiotherapy (refused surgery) groups after PSM

Survival outcomes were also compared between matched patients of those who subjected to surgery versus those subjected to radiotherapy who were recommended to surgery but refused. The demographics and clinical variables were well matched after propensity score matching (Additional file 1: eTable 2). Kaplan–Meier survival curves for the matched groups are displayed in Fig. 5. No differences in the OS (*P* = 0.104) and CSS (*P* = 0.414) were found between the two groups.

## Discussion

The present study showed that compared with surgery, octogenarians treated with radiotherapy were older, later diagnosed, and had more often unmarried, more squamous cell carcinoma, unknown grade and increased tumor size. Younger age, female sex, later year of diagnosis, adenocarcinoma histology, well or moderately differentiation, smaller tumor size and receiving surgery were independent prognostic factors for better OS. Surgery with regional LNE offers better survival than radiotherapy in octogenarians with clinical stage Ia NSCLC after PSM. The 1-, 3-, and 5-year OS rates of surgery and radiotherapy group were 90.0%, 76.9%, 59.9%, and 86.0%, 54.3%, 28.0%, respectively. The 1-, 3-, and 5-year CSS rates of surgery and radiotherapy group were 94.5%, 86.1%, 78.0% and 90.7%, 74.5%, 61.0%, respectively.

The results of our study are consistent with those of most studies and meta-analyses comparing the clinical outcomes of surgery versus radiotherapy in elderly or non-elderly patients [7–10]. However, these studies span a long time, from 1998 to 2018, during which techniques of radiotherapy and surgery developed rapidly, and the results may not be able to guide current medical decisions. We selected the cases in the last 10 years (2012–2017) to eliminate the influence of unbalanced development of technology in radiotherapy and surgery, so that the results can better guide the current clinical decision. We advocate that although the popularity of radiotherapy in elderly patients and the great progress in radiotherapy in the last 10 years, surgery should still be the first choice for patients with stage Ia NSCLC, in view of the better OS and CSS, even for the elderly over 80 years old. As the conclusion of a single-center study [11], age is no reason to deny patients surgery for early-stage lung cancer. Studies [12] have also found that quality of life among octogenarians after surgery remains similar to younger patients even after anatomical lung resection. Though there is still controversy about which operation procedure is the best. Most patients in the surgery group in our study received either lobectomy (63.4%) or wedge resection (27.5%). Chan et al. [13] believes that lobectomy provided better 5-year survival compared with sublobar resection regardless of the age in octogenarians with pathologic stage I lung cancer. Yet the JACS1303 study [14] showed that wedge resection might be equivalent to lobectomy or segmentectomy in selected octogenarians or older with early-stage NSCLC who can tolerate lobectomy. Study from Mimae et al. [15] showed that the influence of wedge resection on death due to other causes was lower than that of lobectomy or segmentectomy in patients with NSCLC aged ≥ 80 years. We speculate that a multidisciplinary team discussion on performance status, cardio-pulmonary function, and so on, may be helpful to choose the surgical procedure.

It is worth mentioning that there were no survival advantages when surgery without LNE compared to matched radiotherapy in the subgroup analysis. An analysis of the National Cancer Database (NCDB) also showed no survival differences between the lobectomy with 0 LN and SABR among healthy patients with clinical stage I NSCLC [16]. We speculate that the patients with stage Ia in surgery with LNE group was “net stage Ia”, while the stage Ia in radiotherapy and surgery without LNE group were not, and patients with lymph node metastasis might be included in these group. There are two inspirations from these results. First, for the surgeons, it is crucial to choose appropriate patient who can tolerate the surgery and perform regional LNE to ensure better survival benefits; Secondly, the disadvantage of radiotherapy compared to surgery is the impossibility of regional lymph node dissection, which may leave out occult lymph node metastasis and affect the overall prognosis [17]. Larger tumors are known to be associated with higher rates of occult nodal involvement [18, 19]. In order to reduce the influence of occult lymph node metastasis as much as possible, patients with a tumor size more than 3 cm were excluded in our study. However, the rate of occult lymph node involvement was 12.9% even in less than 3 cm NSCLC [19]. For radiologists, it is important to perform accurate clinical staging before radiotherapy. Conventional imaging examination such as enhanced computed tomography (CT) or ^18^F-fluorodeoxyglucose positron emission tomography (PET-CT) [20–22] may not be able to judge the nature of lymph nodes accurately. Use of endobronchial ultrasound bronchoscopy (EBUS) with hilar and mediastinal nodal fine-needle aspiration sampling may provide more information on lymph node staging [23, 24]. Recently, the revised STARS study, in which pathological nodal sampling using EBUS was performed in > 90% patients, showed that the 3-year OS rate of SABR and surgical group (video-assisted thoracoscopic surgical lobectomy with mediastinal lymph node dissection, VATS L-MLND) was 91%, the 5-year OS rate in SABR group was 87%, and 84% in surgical group. Long-term survival after SABR was non-inferior to VATS L-MLND for operable stage Ia NSCLC [25]. One may be able to expect better survival outcomes of radiotherapy in early stage NSCLC by accurate lymph node staging.

The findings of this study should not be interpreted as a comparative analysis of surgery and radiotherapy yet, since it may magnify the survival difference between radiotherapy and surgery. Although the outcomes were significantly lower in patients undergoing radiotherapy, these patients were vast majority (91.2%) nonsurgical candidates, that is, these patients were generally with poor physical status, and might have a shorter life expectancy. Bei et al. [26] demonstrated that pretreatment physical state was significantly associated with OS in patients > 80 years old with early-stage NSCLC receiving SABR. Watanabe et al. also revealed that inoperability was the predictors of poor overall survival after SABR in elderly patients [27]. Although we performed PSM to adjust the two groups as much as possible, the lack of data about patient’s performance status and comorbid conditions in SEER database makes it impossible to match completely. This was confirmed by further comparison of outcomes between matched patients of surgery versus radiotherapy who were recommended to surgery but refused. There were no survival advantages when surgery compared to radiotherapy in matched operable stage Ia patients. It is also worth mentioning that not all radiotherapy in SEER database were SABR. As we know, there were significant survival differences between conventional fractionated radiotherapy and SABR [28, 29], which may be one of the reasons for the survival advantages of surgery to radiotherapy in our study. At present, SABR has become the standard radiotherapy modality for inoperable early lung cancer. The revised STARS study selected operable Ia NSCLC and showed that survival of SABR was non-inferior than that of VATS L-MLND [25]. A propensity score‑matching analysis from Tomita et al., in which performance status, forced expiratory volume and Charlson comorbidity index between groups were all matched, also demonstrated that survivals were not significantly different between surgery and SBRT in patients with c-stage I NSCLC [30]. One may infer that survival difference between surgery and radiotherapy may not be as great as in our study among operable patients or patients with good performance status. Therefore, larger RCT studies are required to further clarify which is better for octogenarians with early stage NSCLC. Studies such as STABLE-MATES trial [31] are already under way, and we look forward to the results.

There are several limitations in this study. First, given the retrospective nature of the data collection from the SEER database, inherent selection bias was inevitable. Second, prognostic factors of NSCLC, such as the performance status, comorbid conditions and specific details regarding radiotherapy regimens were not provided by the SEER database. Although propensity score matching analysis was performed, biases cannot be excluded. Prospective trials are required to confirm the findings reported here.

In summary, our study demonstrates that surgery with lymph node dissection is associated with better OS and CSS than radiotherapy in octogenarians and older with stage Ia NSCLC. Our results should be interpreted with caution given the limitations discussed above. More research is needed to determine which is better for stage Ia NSCLC in patients who are operable or have good performance status and patients with accurate lymph node staging.

## Supplementary Information

**Additional file 1** **eTable 1**. Characteristics of patients receiving surgery without LNE and radiotherapy after PSM.** eTable 2**. Characteristics of patients in the surgery and radiotherapy (refused surgery) groups after PSM

## Data Availability

All data in this paper are from SEER database. All data discussed in the manuscript are included within this published article. This data can be found here: https://seer.cancer.gov/.com

## Abbreviations

OS: Overall survival
CSS: Cancer-specific survival
NSCLC: Non-small cell lung cancer
SEER: Surveillance, Epidemiology, and End Results database
PSM: Propensity score‑matching
LNE: Lymph node examination
RT: Radiotherapy
SABR: Stereotactic ablative radiotherapy
