# Supplementary material for: Surgery versus radiotherapy in octogenarians with stage Ia non‑small cell lung cancer: propensity score matching analysis of the SEER database
Source: BMC Pulm Med. 2022 Nov 10;22:411. doi: 10.1186/s12890-022-02177-7 (PMC9650884; doi:10.1186/s12890-022-02177-7)
Supplement: Supplementary file 1 — Additional file 1 eTable 1. Characteristics of patients receiving surgery without LNE and radiotherapy after PSM. eTable 2. Characteristics of patients in the surgery and radiotherapy (refused surgery) groups after PSM [file 12890_2022_2177_MOESM1_ESM.docx]

**Online Resource**

**eTable 1.** Characteristics of patients receiving surgery without LNE and radiotherapy after PSM

| Characteristics | No. (%) | | *p* Value |
| --- | --- | --- | --- |
|  | Surgery with no LNE  (n = 81) | Radiotherapy  (n = 81) |  |
| Age, median ± SD, y | 83.3 ± 2.8 | 83.2 ± 2.7 | 0.842 |
| Sex |  |  | 0.186 |
| Male | 32(39.5%) | 24(29.6%) |  |
| Female | 49(60.5%) | 57(70.4%) |  |
| Race |  |  | 0.667 |
| White | 72(88.9%) | 69(85.2%) |  |
| Black | 2(2.5%) | 4(4.9%) |  |
| Other | 7(8.6%) | 8(9.9%) |  |
| Marital status |  |  | 0.529 |
| Unmarried | 42(51.9%) | 49(60.5%) |  |
| Married | 37(45.7%) | 30(37.0%) |  |
| Unknown | 2(2.5%) | 2(2.5%) |  |
| Year of diagnosis |  |  | 0.530 |
| 2012-2014 | 39(48.1%) | 43(53.1%) |  |
| 2015-2017 | 42(51.9%) | 38(46.9%) |  |
| Tumor size, cm |  |  | 0.815 |
| T ≤ 1 cm | 9(11.1%) | 8(9.9%) |  |
| 1 < T ≤ 2 cm | 46(56.8%) | 50(61.7%) |  |
| 2 < T ≤3 cm | 26(32.1%) | 23(23.4%) |  |
| Histology |  |  | 0.557 |
| Adenocarcinoma | 60(74.1%) | 65(80.2%) |  |
| Squamous cell carcinoma | 17(21.0%) | 14(17.3%) |  |
| Other | 4(4.9%) | 2(2.5%) |  |
| Grade |  |  | 0.193 |
| Well differentiated | 20(24.7%) | 27(33.3%) |  |
| Moderately differentiated | 36(44.4%) | 26(32.1%) |  |
| Poorly differentiated or undifferentiated | 18(22.2%) | 15(18.5%) |  |
| Unknown | 7(8.6%) | 13(16.0%) |  |

**eTable 2.** Characteristics of patients in the surgery and radiotherapy (refused surgery) groups after PSM

| Characteristics | No. (%) | | *p* Value |
| --- | --- | --- | --- |
|  | Surgery  (n = 72) | Radiotherapy  (refused surgery)  (n = 72) |  |
| Age, median ± SD, y | 83.8 ± 3.1 | 84.1.2 ± 2.8 | 0.516 |
| Sex |  |  | 0.374 |
| Male | 21(29.2%) | 26(36.1%) |  |
| Female | 51(70.8%) | 46(63.9%) |  |
| Race |  |  | 0.640 |
| White | 59(81.9%) | 62(86.1%) |  |
| Black | 7(9.7%) | 4(5.6%) |  |
| Other | 6(8.3%) | 6(8.3%) |  |
| Marital status |  |  | 0.282 |
| Unmarried | 42(58.3%) | 51(70.8%) |  |
| Married | 29(40.3%) | 20(27.8%) |  |
| Unknown | 1(1.4%) | 1(1.4%) |  |
| Year of diagnosis |  |  | 1.000 |
| 2012-2014 | 26(36.1%) | 26(36.1%) |  |
| 2015-2017 | 46(63.9%) | 46(63.9%) |  |
| Tumor size, cm |  |  | 0.694 |
| T ≤ 1 cm | 4(5.6%) | 2(2.8%) |  |
| 1 < T ≤ 2 cm | 30(41.7%) | 30(41.7%) |  |
| 2 < T ≤3 cm | 38(52.8%) | 40(55.6%) |  |
| Histology |  |  | 0.315 |
| Adenocarcinoma | 50(69.4%) | 46(63.9%) |  |
| Squamous cell carcinoma | 15(20.8%) | 22(30.6%) |  |
| Other | 7(9.7%) | 4(5.6%) |  |
| Grade |  |  | 0.268 |
| Well differentiated | 9(12.5%) | 5(6.9%) |  |
| Moderately differentiated | 18(25.0%) | 24(33.3%) |  |
| Poorly differentiated or undifferentiated | 20(27.8%) | 13(18.1%) |  |
| Unknown | 25(34.7%) | 30(41.7%) |  |
